# The impact of the COVID-19 pandemic on racial disparities in patients undergoing total shoulder arthroplasty in the United States

**DOI:** 10.1016/j.jseint.2022.10.014

**Published:** 2022-11-12

**Authors:** The Avant-garde Health and Codman Shoulder Society Value Based Care Group, Matthew J. Best, Catherine J. Fedorka, Robert M. Belniak, Derek A. Haas, Xiaoran Zhang, April D. Armstrong, Joseph A. Abboud, Andrew Jawa, Evan A. O’Donnell, Jason E. Simon, Eric R. Wagner, Momin Malik, Michael B. Gottschalk, Adam Z. Khan, Gary F. Updegrove, Eric C. Makhni, Jon J.P. Warner, Uma Srikumaran

**Affiliations:** a Department of Orthopaedic Surgery, Johns Hopkins University School of Medicine, Johns Hopkins Hospital, Baltimore, MD, USA; b Cooper Bone and Joint Institute, Camden, NJ, USA; c Department of Orthopaedic Surgery and Sports Medicine, Starling Physicians Group, New Britain, CT, USA; d Avant-garde Health, Boston, MA, USA; e Department of Orthopaedics and Rehabilitation, Bone and Joint Institute, Penn State Milton S. Hershey Medical Center, Hershey, PA, USA; f Rothman Institute, Thomas Jefferson University Hospital, Philadelphia, PA, USA; g Department of Orthopaedic Surgery, New England Baptist Hospital, Tufts University School of Medicine, Boston, MA, USA; h Boston Sports and Shoulder Center, Waltham, MA, USA; i Department of Orthopaedic Surgery, Harvard Medical School, Massachusetts General Hospital, Boston Shoulder Institute, Boston, MA, USA; j Department of Orthopaedic Surgery, Harvard Medical School, Newton-Wellesley Hospital, Boston, MA, USA; k Department of Orthopaedic Surgery, Emory University, Atlanta, GA, USA; l Northwest Permanente Physicians and Surgeons, Clackamas, OR, USA; m Department of Orthopaedic Surgery, Sports Medicine, Henry Ford Health, Detroit, MI, USA

**Keywords:** Racial disparities, COVID-19, Coronavirus pandemic, Total shoulder arthroplasty, TSA, Shoulder

## Abstract

**Introduction:**

The purpose of this study was to assess racial disparities in total shoulder arthroplasty (TSA) in the United States and to determine whether these disparities were affected by the COVID-19 pandemic.

**Methods:**

Centers for Medicare and Medicaid Services (CMS) 100% sample was used to examine primary TSA volume from April to December from 2019 to 2020. Utilization was assessed for White, Black, Hispanic, and Asian populations to determine if COVID-19 affected these groups differently. A regression model adjusted for age, sex, CMS-hierarchical condition categories (HCC) score, dual enrollment (proxy for socioeconomic status), time-fixed effects, and core-based statistical area fixed effects was used to study difference across groups.

**Results:**

In 2019, the TSA volume per 1000 beneficiaries was 1.51 for White and 0.57 for non-White, with a 2.6-fold difference. In 2020, the rate of TSA in White patients (1.30/1000) was 2.9 times higher than non-White (0.45/1000) during the COVID-19 pandemic (*P* < .01). There was an overall 14% decrease in TSA volume per 1000 Medicare beneficiaries in 2020; non-White patients had a larger percentage decrease in TSA volume than White (21% vs. 14%, estimated difference; 8.7%, *P* = .02). Black patients experienced the most pronounced disparity with estimated difference of 10.1%, *P* = .05, compared with White patients. Similar disparities were observed when categorizing procedures into anatomic and reverse TSA, but not proximal humerus fracture.

**Conclusions:**

During the COVID-19 pandemic, overall TSA utilization decreased by 14% with White patients experiencing a decrease of 14%, and non-White patients experiencing a decrease of 21%. This trend was observed for elective TSA, while disparities were less apparent for proximal humerus fracture.

Racial disparities have been shown in many areas of medicine and have persisted through the past decade.^9^^,^^12^^,^^15^^,^^24^^,^^25^ Achieving health equity and improving access to quality medical care for underrepresented minorities has been a focus for medical societies, patient advocacy groups, and various areas of government, prompting the development of federal programs and policies aimed at reducing health inequality.^3^, ^4^, ^5^, ^6^^,^^20^ Despite knowledge of racial disparities and the implementation of various policies and methods to reduce them, racial disparities have persisted in the United States for a myriad of surgical procedures including shoulder arthroplasty.^1^^,^^8^

The onset of the COVID-19 pandemic has led to widespread economic and health care crises affecting patients, providers, and the health care system overall.^10^ Several reports have demonstrated that the Black community has been disproportionately affected by COVID-19 both in rates of hospitalization and risk of death.^14^^,^^21^^,^^22^^,^^28^ Price-Haygood et al showed that Black patients with COVID-19 had a significantly higher mortality rate than White patients within a large, tertiary care network.^22^ This finding was particularly problematic given that Black patients made up less than one-third of the population studied. Worsening racial disparities during the COVID-19 pandemic have also been shown for Medicare patients undergoing hip and knee replacement surgery, with a 12.9% decreased likelihood of undergoing hip or knee replacement for non-White patients.^26^

The purpose of this study was to assess the impact that the COVID-19 pandemic has had on racial disparities in patients undergoing total shoulder arthroplasty (TSA). We also aim to evaluate these disparities in the context of indication for shoulder arthroplasty, comparing those treated for proximal humerus fracture with nonfracture cases. We hypothesize that shoulder arthroplasty utilization for all races decreased during the COVID-19 pandemic but that a greater decrease occurred in Black, Hispanic, and Asian patients, compared with White patients.

## Methods

### Data source

The Center for Medicare and Medicaid Services (CMS) fee-for-service inpatient and outpatient claims data of 100% sample, and Medicare enrollment data spanning 2019 through 2020 were used for this analysis. We identified April 2020 as the first full month after the onset of the COVID-19 pandemic, and therefore set April 1, 2020 as the start date for the COVID-19 period. Since at the time of our analysis, the claims data extended through December 31, 2020, we examined cases admitted by December 18th to ensure we could capture the full length of stay. We examined the same time period of cases (April 1st to December 18th admissions) in 2019 as in 2020 to ensure comparability between the years. The study included primary total shoulder arthroplasties (TSAs) that were coded as inpatient using diagnosis-related group 483, and outpatient using primary Current Procedural Terminology (CPT) 23472. Outpatient TSAs only made up only a small fraction of total TSAs cases as CMS only removed TSAs from the inpatient-only list in 2021. We further divided TSAs into nonfracture anatomic arthroplasties, nonfracture reverse arthroplasties, and fracture TSAs (anatomic and reverse combined) based on each case’s primary international classification of diseases (ICD) 10th revision diagnosis (ICD-10-CM) and procedure (ICD-10-PCS) codes. CMS previously expanded their variables for non-White and non-Black race and ethnicity groups to allow for more granular data collection. Therefore, we analyzed racial and ethnic groups individually (White, Black, Hispanic, and Asian) as well as in larger groups (White and non-White).

### Race/ethnicity

We defined race and ethnicity using the race variable from the Medicare beneficiary enrollment data. Race and ethnicities include White, Black, Hispanic, Asian, other, and unknown. We grouped minorities including Black, Hispanic, Asian, and other into the non-White racial group to compare with the White racial group. We also compared Black, Asian, Hispanic, and other minority groups with White separately.

### Statistical analysis

We calculated the total TSA volume per 1000 Medicare beneficiaries by different racial groups. We then compared the total TSA rate between April 1st and December 18th in 2019 and 2020. The percentage change in TSA rate during COVID-19 was then calculated. A generalized linear model assuming a binomial distribution at beneficiary year-level was fitted with the dependent variable being if a beneficiary received a TSA between April and December during the given year. We adjusted for the minority indicator that interacted during COVID-19, age, sex, CMS-hierarchical condition categories (HCC) risk score, Medicare-Medicaid dual enrollment status, and year fixed effects, to study the differential effect of COVID-19 on operation rate changes across different racial populations. Bonferroni corrections were applied to *P*-values. We controlled for the CMS-HCC risk score because it is a measure reflecting the expected future health costs for each patient based on the patient demographics and chronic illnesses. Medicare-Medicaid dual enrollment, i.e. if a patient is enrolled in both Medicare and Medicaid, was controlled for as a proxy for economic status of the beneficiary since the eligibility is income-based. The coefficient of the interaction term between minorities and COVID-19 is our estimate of the impact of the COVID-19 pandemic on racial disparities between White and the minority racial population. This shows how much the TSA operation rate changed due to COVID-19 in the minority racial populatio, compared to the White racial group.

## Results

A total of 49,412 and 41,554 cases were observed in 2019 and 2020 between April and December, respectively. There has been an overall 14% decrease in TSA volume per 1000 Medicare beneficiaries between 2019 and 2020 (1.51 vs. 1.30). Racial disparities existed for TSA nationally prior to the COVID-19 pandemic. In 2019, the TSA hospitalization volume per 1000 Medicare beneficiaries was 1.69 for the White population and 0.57 for the non-White population, with a 2.6-fold difference. In 2020, the White TSA hospitalization rate (0.45 per 1000 beneficiaries) was 2.9 times higher than that of the non-White patients (0.45 per 1000 beneficiaries) during the COVID-19 pandemic. The percentage decrease between 2019 and 2020 for White and non-White population was 14% and 21%, respectively (Table I).

**Table I:** Changes in rates of TSAs per 1000 Medicare beneficiaries by racial group during the COVID-19 pandemic.

	2019 Apr-Dec	2020 Apr-Dec	Change (%)
All TSAs (sum of types below)	1.51	1.30	−14%
White	1.69	1.46	−14%
Non-White	0.57	0.45	−21%
Black	0.58	0.45	−22%
Hispanic	0.50	0.42	−16%
Asian	0.29	0.22	−25%
Other minorities	0.82	0.68	−17%
Unknown	1.31	1.16	−11%
Types of hospitalizations			
Anatomic TSA non-fracture	0.45	0.36	−19%
White	0.50	0.40	−19%
Non-White	0.16	0.11	−29%
Black	0.18	0.12	−29%
Hispanic	0.11	0.08	−31%
Asian	0.07	0.05	−35%
Other minorities	0.21	0.16	−23%
Unknown	0.51	0.45	−11%
Reverse TSA nonfracture	0.91	0.80	−13%
White	1.02	0.89	−13%
Non-White	0.36	0.29	−19%
Black	0.38	0.30	−20%
Hispanic	0.32	0.28	−12%
Asian	0.17	0.12	−30%
Other minorities	0.53	0.44	−17%
Unknown	0.72	0.64	−12%
Anatomic + fracture TSA fracture	0.15	0.15	−3%
White	0.17	0.17	−3%
Non-White	0.04	0.04	−4%
Black	0.03	0.03	−5%
Hispanic	0.06	0.06	−11%
Asian	0.05	0.05	9%
Other minorities	0.08	0.08	−6%
Unknown	0.07	0.07	−4%

*TSA*, total shoulder arthroplasty.

Similar trends were observed when breaking the arthroplasties into anatomic and reverse TSAs. For anatomic TSAs, the overall decrease was 19% between 2019 and 2020, with the White populating decreasing by 19% and the non-White populating decreasing by 29%. For reverse TSAs, a 13% decrease was observed across populations between 2019 and 2020, with a 13% decrease for the White population and 19% decrease for the non-White population. Disparities were less apparent when analyzing nonelective cases, with a 3% decrease overall, a 3% decrease among White patients compared with a 4% decrease for non-White patients undergoing shoulder arthroplasty for proximal humerus fracture (Table I).

By fitting a logistic regression at beneficiary year-level with receiving a TSA as dependent variable, we were able to quantify the impact of COVID-19 and race combined. Table II shows the estimate of the interaction term between COVID-19 and minorities, quantifying the exacerbation of preexisting racial disparities due to COVID-19. When pooling all non-White patients, the estimated difference in TSA utilization compared with White patients was −8.7% (95% confidence interval [−14.0%, −3.1%], *P* = .02), meaning that with TSA utilization decreasing for both White and non-White populations, there was an 8.7% more decrease for the non-White population. This effect was especially pronounced for the Black population, with a −10.1% (95% confidence interval [−16.8%, −2.9%], *P* = .05) estimated difference compared with White. For other minorities, however, we did not observe significant differences according to the regression.

**Table II:** Estimated between-group differences of TSA rates for all types of TSAs for each minority group compared with the White population∗.

	Estimate	95% CI	*P* value
Non-White vs. White	−8.7%	(−14.0%, −3.1%)	.02
Black vs. White	−10.1%	(−16.8%, −2.9%)	.05
Hispanic vs. White	−2.6%	(−17.3%, 14.8%)	1.00
Asian vs. White	−18.2%	(−34.7%, 2.4%)	.64
Other Minorities vs. White	−4.5%	(−15.7%, 8.3%)	1.00

*CI*, confidence interval; *CMS-HCC*, Centers for Medicade and Medicare Services hierarchal condition categories risk score; *TSA*, total shoulder arthroplasty.

∗ Estimated between-group differences for operation rate were made from the logistic regression model at beneficiary-year level, adjusted for age, sex, CMS-HCC, risk score, and Medicare-Medicaid dual enrollment status. Bonferroni corrections were applied to *P*-values. Coefficients were converted as the percentage differences in probability of receiving a TSA, for minorities compared to White. The comparisons were made based on April through December data for 2020 and 2019.

## Discussion

This study showed that after the onset of the COVID-19 pandemic, preexisting racial disparities in patients undergoing TSA worsened. The overall rate of TSA hospitalization decreased by 14% across racial groups during the COVID-19 pandemic. However, the White population experienced a decrease of 14%, compared with a decrease of 21% in the non-White population. When analyzing disparities by indication for surgery, disparities were less pronounced in patients undergoing arthroplasty for proximal humerus fracture, with a decrease in the rate of 3% and 4% for White and non-White patients, respectively. After controlling for age, sex, comorbidities, and dual enrollment status (a proxy for economic status), the estimated difference in percent decrease between White and non-White patients was 8.7%, indicating that the non-White population had an 8.7% larger decrease in the rate of hospitalization than White patients. The estimate was 10.1% for the Black patients.

Racial disparities have been shown in many studies throughout the orthopedic and general medical literature.^1^^,^^8^^,^^9^^,^^12^^,^^15^^,^^24^^,^^25^ This is particularly concerning in the arthroplasty literature since Black patients develop osteoarthritis at a prevalence equal to or greater than White patients.^16^^,^^27^ In addition, various initiatives and programs have been implemented by the American Academy of Orthopedic Surgeons and other advocacy groups to reduce or eliminate differences in musculoskeletal care based on race.^3^^,^^20^ The Musculoskeletal Health care Disparities Research Symposium addressed this issue and called for increased access to arthroplasty procedures for under-represented minority patients.^20^ Despite these initiatives and what seems to be widespread knowledge that these disparities exist, several studies show that racial disparities have persisted through 2017 for many orthopedic procedures and have worsened for some surgeries such as TSA, total hip arthroplasty and total knee arthroplasty (TKA).^1^^,^^2^^,^^8^^,^^9^^,^^19^^,^^23^ In a national study assessing all-payer data from 2006 to 2015, Amen et al showed that there were persistent disparities in utilization between White and Black patients undergoing TKA and worsening racial disparities in complications following TKA.^1^ In a study evaluating racial disparities in patients undergoing TSA, Black patients had lower rates of utilization, higher rates of complications, and increased odds of mortality following surgery compared with White patients.^8^ This lower rate of TSA utilization by Black patients worsened over the study period from 2012-2017.^8^

Racial differences were less apparent in patients undergoing arthroplasty for proximal humerus fracture with a decrease of 3% in White patients and 4% in non-White patients during the pandemic. Many shoulder arthroplasty cases performed for proximal humerus fracture are not elective surgeries and the difficulty in access to subspecialty orthopedic care may not be as marked in the trauma setting. However, racial disparities have been shown in multiple studies for patients undergoing fracture care.^2^^,^^19^^,^^23^ In a study of over 40,000 patients admitted to US hospitals with a proximal humerus fracture, Hispanic and Black patients were more likely to be uninsured and less likely to use postdischarge support services than White patients.^18^ Two national studies assessed racial disparities in hip fracture care and showed longer time to surgery and higher rates of complications in under-represented minority patients compared with White patients.^2^^,^^19^

Several reports and major news outlets have highlighted the glaring racial disparities exacerbated by the COVID-19 pandemic.^14^^,^^21^^,^^22^^,^^26^^,^^28^ In a large cohort of patients within an integrated delivery health care system, 77% of patients hospitalized with COVID-19 and 71% of those who died were Black despite the fact that Black patients comprised only 31% of the health system population in the study.^22^ These findings were corroborated in another study which showed that 34% of COVID-19 deaths were among Black patients despite this group accounted for only 12% of the population in the United States.^14^ These studies highlight that the COVID-19 pandemic has magnified another pandemic in the United States, which is racial and ethnic disparities in health care.^29^

There are several explanations for the findings of this study which have been posited as reasons for health care disparities throughout the pandemic. Access to primary care and specialty care has been shown to be more difficult for under-represented minorities.^21^^,^^29^ Implicit bias is associated with worse quality of care and poorer communication between patients and physicians.^13^^,^^17^^,^^29^ Bias in behavioral attitudes can be worsened under conditions of stress such as providing medical care during the COVID-19 pandemic.^29^ This may be reflected in data from various regions in the United States which showed that African American patients with cough or fever were less likely than White patients to be offered a COVID test.^7^ Another factor contributing to racial disparities which may be exacerbated during the COVID-19 pandemic is pathogenic effects of adverse living and working conditions.^21^ Data from New York City showed that the Bronx had the lowest income and education levels, the highest population of under-represented minorities, and the highest rate of COVID-19 hospitalizations and deaths, despite a lower population density than Manhattan.^28^ Finally, distrust in the medical system has been shown as a factor contributing to racial disparities in patients contemplating total knee replacement and may influence decisions for patients considering total shoulder replacement as well.^11^

Although the results of the present study do not provide reasons for the worsening racial disparities in patients undergoing TSA during the COVID-19 pandemic, they do highlight and raise awareness of this important issue. A recent viewpoint by Yancy recommends three strategies for helping to reduce racial disparities in health care, the first of which is to raise awareness of the problem.^30^ Another strength of this study is that many factors such as age, sex, and economic status, or dual-enrollment status, were controlled for in our regression analysis. Although we do not have specific socioeconomic variables such as household income, dual enrollment status is based on income and can be used to as an indicator of socioeconomic status.

Despite these strengths this study has several limitations. One, we were not able to study these racial trends across different geographic regions or census divisions. Since the overall incidence of TSA in the Medicare population is < 2 per 1000 beneficiaries, this did not provide sufficient sample size for accurate census division analysis. In addition, the patient population studied included Medicare beneficiaries but not private payers. This may have been beneficial in this study since a larger proportion of patients with private insurance may have undergone outpatient TSA. Among Medicare beneficiaries in our study period from 2019 to 2020, the percentage of outpatient TSA was just 1.4%, indicating a large catchment of our study sample.

## Conclusion

COVID-19 exacerbated the preexisting racial disparities for TSA utilization among Medicare beneficiaries in the United State. During the COVID-19 pandemic, the overall TSA hospitalization rate dropped by 14% across racial groups. However, COVID-19 impacted racial groups differently, with the White population experiencing a decrease of 14%, and the non-White population experiencing a decrease of 21%. This trend was observed for elective shoulder arthroplasty cases while disparities were less apparent in patients undergoing arthroplasty for proximal humerus fractures.

## Disclaimers

Funding: No financial support was used for this manuscript.

Conflicts of interest: Matthew J. Best: Reports other financial or material support from Arthrex, Inc and Smith & Nephew. Catherine J. Fedorka, MD: Reports personal fees from Stryker. Robert M. Belniak, MD: Reports presenter fees from Pacira and ownership of stock in Stryker. Derek A. Haas, MBA: Reports ownership of stock in Avant-gard Health and personal fees from Medacta. April D. Armstrong, MD: Reports personal fees from Globus Medical, IP royalties from Zimmer and membership on the editorial or governing board of Journal of Shoulder and Elbow Surgery. Joseph A. Abboud, MD: ownership of stock in Aevumed, research support from Arthrex, personal fees from Bioventus, research support from the Department of Defense, IP royalties and personal fees from DJ Orthopaedics, IP royalties and personal fees Globus Medical, membership on the editorial or governing board of Journal of Shoulder and Elbow Surgery, membership on the editorial or governing board of research support from Lima, ownership of stock in Marlin Medical Alliance, ownership of stock in OBERD, research support from OREF, research support from Orthofix, IP royalties from OsteoCentric Technologies. ownership of stock in OTS Medical, ownership of stock in Shoulder JAM, financial or material support from SLACK Incorporated, IP royalties from Smith and Nephew, IP royalties and personal fees from Stryker, publishing royalties and financial or material support from Wolters Kluwer Health – Lippincott Williams & Wilkins, IP royalties, personal fees and research support from Zimmer. Andrew Jawa, MD: Reports other financial or material support from Boston Outpatient Surgical Suites, other financial or material support from DePuy, A Johnson & Johnson Company, personal fees and research support from DJ Orthopaedics, IP royalties and ownership of stock from Ignite Orthopaedics, membership on the editorial or governing board of Journal of Shoulder and Elbow Surgery, publishing royalties and other financial or material support from Oberd. Jason E. Simon, MD: This author, their immediate family, and any research foundation with which they are affiliated did not receive any financial payments or other benefits from any commercial entity related to the subject of this article. Eric R. Wagner, MD: Reports personal fees from Acumed, personal fees from Biomet, research support from Konica Minolta, personal fees from Osteoremedies, and personal fees from Stryker. Michael B. Gottschalk, MD: Reports research support from Konica Minolta and research support from Stryker. Eric C. Makhni, MD: Reports ownership of stock in OutcomeMD, ownership of stock in Proterea Health, personal fees from Smith & Nephew and publishing royalties and financial or material support from Springer. Jon JP Warner, MD: Reports membership on the editorial or governing board of Journal of Shoulder and Elbow Surgery, other financial or material support from Smith & Nephew, personal fees from Stryker and IP royalties and personal fees from Wright Medical Technology. Uma Srikumaran, MD, MBA, MPH: Reports grants and personal fees from Arthrex, grants and personal fees from Depuy, personal fees from Fx Shoulder, personal fees from Orthofix, other from Quantum OPS, other from ROM3, grants from Smith and Nephew, other from Sonogen, personal fees from Stryker, personal fees from Thieme, personal fees and other from Tigon Medical, grants and personal fees from Wright Medical Technology, outside the submitted work; In addition, Dr. Srikumaran has a patent Conventus pending, a patent Fx Shoulder pending, and a patent Tigon Medical issued. The other authors, their immediate families, and any research foundation with which they are affiliated have not received any financial payments or other benefits from any commercial entity related to the subject of this article. Institutional review board approval was not required for this study.

## References

[1] Amen T.B., Varady N.H., Rajaee S., Chen A.F. (2020). Persistent racial disparities in utilization rates and Perioperative metrics in total joint arthroplasty in the U.S.: a comprehensive analysis of trends from 2006 to 2015. J Bone Joint Surg Am.

[2] Amen T.B., Varady N.H., Shannon E.M., Chopra A., Rajaee S., Chen A.F. (2022). Racial and ethnic disparities in hip fracture surgery care in the United States from 2006 to 2015: a nationwide trends study. J Am Acad Orthop Surg.

[3] Anon (2019). https://www.movementislifecaucus.com/wp-content/uploads/Movement-Is-Life-A-Catalyst-For-Change-Proceedings-Report.pdf.

[4] Anon (2006). https://www.nimhd.nih.gov/docs/2002_2006__vol1_031003ed_rev.pdf.

[5] Anon. U.S. Department of Health and Human Services (2011). HHS action plan to reduce racial and ethnic disparities: a nation free of disparities in health and health care. http://www.minorityhealth.hhs.gov/npa/files/Plans/HHS/HHS_Plan_complete.pdf.

[6] Anon, Wyatt R., Laderman M., Botwinick L., Mate K., Whittington J. (2016). http://ihi.org.

[7] Farmer B., Anon (2020). The coronavirus doesn’t discriminate, but U.S. health care showing familiar biases. NPR. https://www.npr.org/sections/health-shots/2020/04/02/825730141/the-coronavirus-doesntdiscriminate-but-u-s.

[8] Best M.J., Aziz K.T., McFarland E.G., Martin S.D., Rue J.P.H., Srikumaran U. (2021). Worsening racial disparities in patients undergoing anatomic and reverse total shoulder arthroplasty in the United States. J Shoulder Elbow Surg.

[9] Best M.J., McFarland E.G., Thakkar S.C., Srikumaran U. (2021). Racial disparities in the use of surgical procedures in the US. JAMA Surg.

[10] Blumenthal D., Fowler E.J., Abrams M., Collins S.R. (2020). Covid-19 - implications for the health care system. N Engl J Med.

[11] Chang H.J., Mehta P.S., Rosenberg A., Scrimshaw S.C. (2004). Concerns of patients actively contemplating total knee replacement: differences by race and gender. Arthritis Rheum.

[12] Goodman S.M., Parks M.L., McHugh K., Fields K., Smethurst R., Figgie M.P. (2016). Disparities in outcomes for African Americans and whites undergoing total knee arthroplasty: a systematic literature review. J Rheumatol.

[13] Hall W.J., Chapman M.V., Lee K.M., Merino Y.M., Thomas T.W., Payne B.K. (2015). Implicit racial/ethnic bias among health care Professionals and its influence on health care outcomes: a systematic review. Am J Public Health.

[14] Holmes L., Enwere M., Williams J., Ogundele B., Chavan P., Piccoli T. (2020). Black-white risk differentials in COVID-19 (SARS-COV2) transmission, mortality and case fatality in the United States: translational epidemiologic Perspective and challenges. Int J Environ Res Public Health.

[15] Jha A.K., Fisher E.S., Li Z., Orav E.J., Epstein A.M. (2005). Racial trends in the use of major procedures among the elderly. N Engl J Med.

[16] Jordan J.M., Helmick C.G., Renner J.B., Luta G., Dragomir A.D., Woodard J. (2007). Prevalence of knee symptoms and radiographic and symptomatic knee osteoarthritis in African Americans and Caucasians: the Johnston County osteoarthritis Project. J Rheumatol.

[17] Maina I.W., Belton T.D., Ginzberg S., Singh A., Johnson T.J. (2018). A decade of studying implicit racial/ethnic bias in healthcare providers using the implicit association test. Soc Sci Med.

[18] Menendez M.E., Ring D. (2014). Racial and insurance disparities in the utilization of supportive care after inpatient admission for proximal humerus fracture. Shoulder Elbow.

[19] Nayar S.K., Marrache M., Ali I., Bressner J., Raad M., Shafiq B. (2020). Racial disparity in time to surgery and complications for hip fracture patients. Clin Orthop Surg.

[20] O’Connor M.I., Lavernia C.J., Nelson C.L. (2011). AAOS/ORS/ABJS Musculoskeletal Healthcare Disparities Research Symposium: editorial comment: a call to arms: eliminating musculoskeletal healthcare disparities. Clin Orthop Relat Res.

[21] Owen W.F., Carmona R., Pomeroy C. (2020). Failing another national stress test on health disparities. JAMA.

[22] Price-Haywood E.G., Burton J., Fort D., Seoane L. (2020). Hospitalization and mortality among black patients and white patients with COVID-19. N Engl J Med.

[23] Raad M., Puvanesarajah V., Wang K.Y., McDaniel C.M., Srikumaran U., Levin A.S. (2022). Do disparities in wait times to operative fixation for Pathologic fractures of the long bones and 30-day complications exist between black and white patients? A study using the NSQIP database. Clin Orthop Relat Res.

[24] Schoenfeld A.J., Tipirneni R., Nelson J.H., Carpenter J.E., Iwashyna T.J. (2014). The influence of race and ethnicity on complications and mortality after orthopedic surgery: a systematic review of the literature. Med Care.

[25] Singh J.A., Lu X., Rosenthal G.E., Ibrahim S., Cram P. (2014). Racial disparities in knee and hip total joint arthroplasty: an 18-year analysis of national Medicare data. Ann Rheum Dis.

[26] Stronach B.M., Zhang X., Haas D., Iorio R., Anoushiravani A., Barnes C.L. (2022). Worsening arthroplasty utilization with widening racial variance during the COVID-19 pandemic. J Arthroplasty.

[27] Vaughn I.A., Terry E.L., Bartley E.J., Schaefer N., Fillingim R.B. (2019). Racial-ethnic differences in osteoarthritis Pain and disability: a meta-analysis. J Pain.

[28] Wadhera R.K., Wadhera P., Gaba P., Figueroa J.F., Joynt Maddox K.E. (2020). Variation in COVID-19 hospitalizations and deaths across New York City boroughs. JAMA.

[29] Williams D.R., Cooper L.A. (2020). COVID-19 and health equity-A new kind of “herd immunity. JAMA.

[30] Yancy C.W. (2020). COVID-19 and African Americans. JAMA.

